# Difference in radiofrequency ablation profile between during sinus rhythm and atrial fibrillation: Considerations in this era of high‐power short‐duration strategy

**DOI:** 10.1002/joa3.13025

**Published:** 2024-03-18

**Authors:** Satoshi Hara, Shigeki Kusa, Yoshikazu Sato, Hiroaki Ohya, Naoyuki Miwa, Hidenori Hirano, Taiki Ishizawa, Tadanori Nakata, Junichi Doi, Hitoshi Hachiya

**Affiliations:** ^1^ Cardiovascular Center, Tsuchiura Kyodo Hospital Tsuchiura Ibaraki Japan

**Keywords:** ablation index, atrial fibrillation, contact force, contact force stability, left atrial function

## Abstract

**Background:**

The concept of ablation index (AI) was introduced to evaluate radiofrequency (RF) ablation lesions. It is calculated from power, contact force (CF), and RF duration. However, other factors may also affect the quality of ablation lesions. To examine the difference in RF lesions made during sinus rhythm (SR) and atrial fibrillation (AF).

**Methods:**

Sixty patients underwent index pulmonary vein isolation during SR (*n* = 30, SR group) or AF (*n* = 30, AF group). All ablations were performed with a power of 50 W, a targeted CF of 5–15 g, and AI of 400–450 using Thermocool Smarttouch SF. The CF, AI, RF duration, temperature rise (Δtemp), impedance drop (Δimp), and the CF stability of each ablation point quantified as the standard deviation of the CF (CF‐SD) were compared between the two groups.

**Results:**

A total of 3579 ablation points were analyzed, which included 1618 and 1961 points in the SR and the AF groups, respectively. Power, average CF, RF duration per point, and the resultant AI (389 ± 59 vs. 388 ± 57) were similar for the two rhythms. However, differences were seen in the CF‐SD (3.5 ± 2.2 vs. 3.8 ± 2.1 g, *p* < .01), Δtemp (3.8 ± 1.3 vs. 4.0 ± 1.3°C, *p* < .005), and Δimp (10.3 ± 5.8 vs. 9.4 ± 5.4 Ω, *p* < .005).

**Conclusions:**

Despite similar AI, various RF parameters differed according to the underlying atrial rhythm. Ablation delivered during SR demonstrated less CF variability and temperature increase and greater impedance drop than during AF.

## Graphical abstract

Differences in the radiofrequency effects were observed during atrial fibrillation (AF) and sinus rhythm (SR). The stability of the contact force and impedance drop was greater during SR, but the temperate rise was greater during AF.

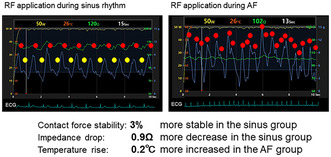

## 1 INTRODUCTION

The ablation index^1^ (AI) was recently introduced as a marker of lesion quality for atrial fibrillation (AF) ablation, and AI‐based high‐power short‐duration (HPSD) protocol pulmonary vein isolation (PVI) has become popular.^2^ Research has found high contact force (CF) to be unnecessary^3^ or even something to be actively avoided^4^ compared to the conventional low‐power long‐duration protocol.^5^ It has been reported that improving the stability of CF reduced the AF recurrence rate after ablation,^6^ and various techniques have been reported to improve the stability, such as using a steerable sheath^7^ or performing rapid ventricular pacing, but there is no report comparing the stability by the underlying rhythm at the time of RF application. AI is calculated using CF, power, and RF duration with a sampling frequency of 100 ms and does not include other factors. We hypothesized that CF stability would be affected by atrial rhythm because CF is influenced by atrial wall movement^8^, ^9^ and, in turn, lead to differences in RF lesion. The purpose of this study was to examine the difference in RF lesions made during sinus rhythm (SR) and atrial fibrillation (AF).

## 2 METHODS

We retrospectively analyzed 30 consecutive patients in SR at the time of ablation (SR group) and 30 patients in AF at the time of ablation (AF group) who underwent initial pulmonary vein isolation (PVI) at our institution by 4 operators (S.K, Y.S, H.O, and S.H). All patients received PVI with a steerable long sheath that can be visualized (VIZIGO; Biosense Webster). This study was approved by the Institutional Review Board of our hospital and adhered to the principles of Declaration of Helsinki.

### 2.1 Ablation procedure

**FIGURE 1:**
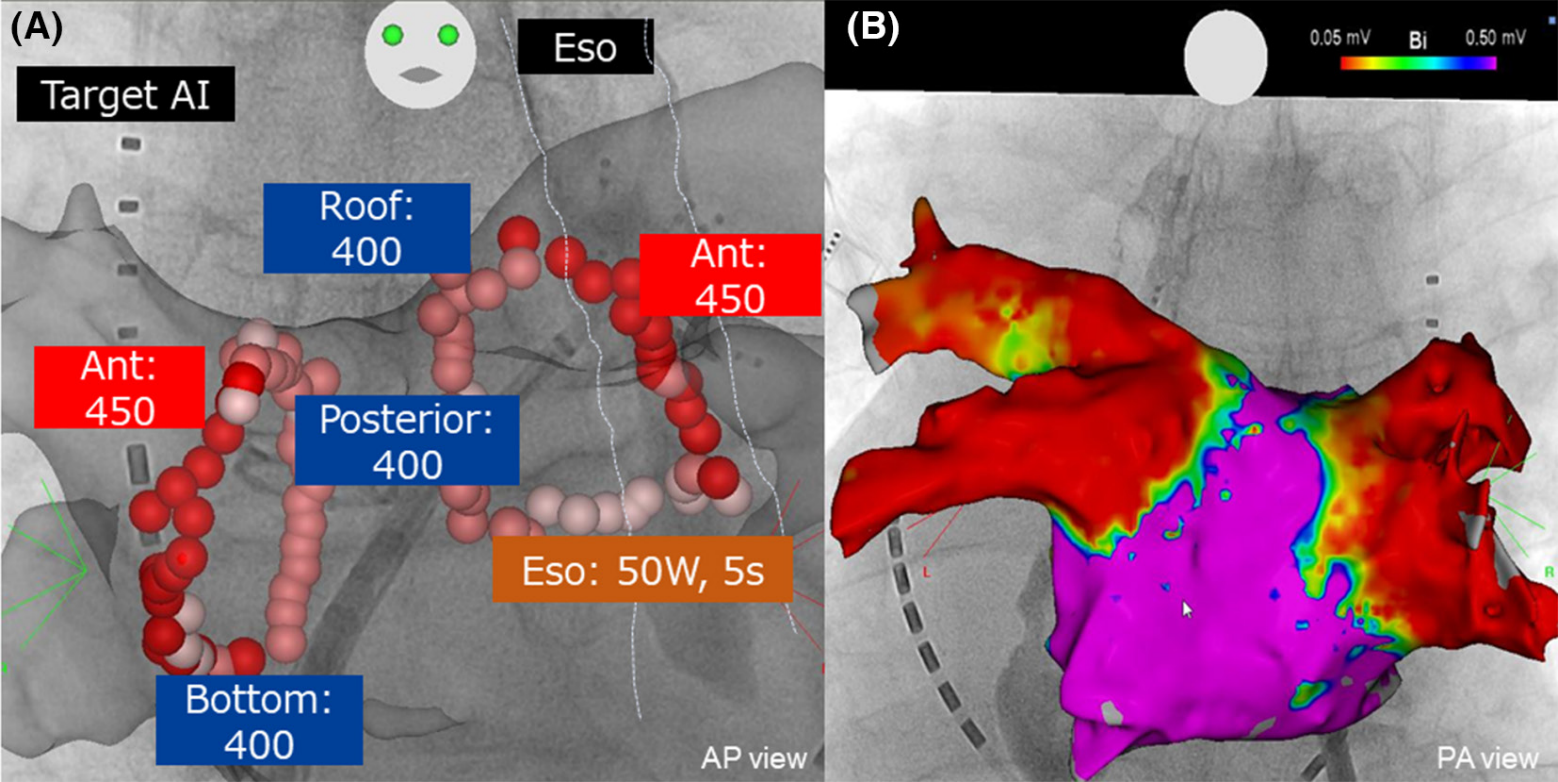
(A) Target AI settings in each PV lesion in this study. Ablation points in the esophagus were excluded from the analysis. (B) The endpoint of PVI. Confirmation of absence of PV potentials greater than 0.05 mV on the voltage map.

Ablation procedures were uniformly performed without mechanical ventilation and under conscious sedation with opioids. The transseptal puncture was performed under intracardiac echo (CARTO Sound; Biosense Webster) with an RF transseptal needle (RF Needle; Baylis Medical). VIZIGO and a nonsteerable long sheath (SL‐1; Abbot) were introduced into the left atrium (LA) via a single transseptal puncture hole. The geometry of the LA was recreated by a multi‐electrode mapping catheter (Pentaray; Biosense Webster) during coronary sinus (CS) pacing in the SR group and during AF rhythm in the AF group. We performed extensive encircling pulmonary vein isolation (EEPVI)^9^ using a 3.5 mm externally irrigated tip catheter with contact force sensing (Thermocool Smarttouch SF; Biosense Webster) and VIZIGO sheath.^7^ The endpoint of each RF application was guided by the AI except for esophagus lesions. Radiofrequency ablation was performed using an AI with a target of 450 for the anterior wall and 400 for other segments with an inter‐lesion distance of 6 mm or less (Figure 1A). In these areas, RF delivery was 50 W and the targeted CF was between 5 and 15 g. For the lesions adjacent to the esophagus in esophagography, a 50 W RF application was delivered for 5 s with a CF between 5 and 10 g. After encircling the PV, internal defibrillation was performed (BeeAT; Japan Lifeline) if AF remained. Voltage maps under CS distal pacing (600 ms) were created by Pentaray, and EEPVI endpoints were confirmed by absence of PV potentials, defined as bipolar voltage less than 0.05 mV in the voltage map. If PV isolation was not achieved, additional RF applications were performed until confirmation of absence of PV potential in the voltage map (Figure 1B). Acute PV reconnection was defined as reconnection after PVI during a waiting period of 20 min or provocation with intravenous adenosine administration.^10^ We defined LA dwelling time as time from LA access by the Brockenbrough procedure to time of confirmation of PV isolation. Concomitant cavotricuspid ablation and/or superior vena cava isolation by high‐power short‐duration protocol^11^ was performed according to the operator's decision.

### 2.2 Evaluation of left atrial function

A preprocedural echocardiogram was performed in all patients. Using Echo PAC version 204 (GE Healthcare), we obtained in addition to the usual parameters such as left atrial diameter and volume, two measures of left atrial strain, LA‐Ss and LA‐Sa. Ss represents the reservoir function of the LA, and Sa represents the conductor function of the LA.

### 2.3 Analysis of ablation data

Parameters of each ablation point were obtained according to the VISITAG module including the mean CF (g), power output (W), RF duration (sec), impedance drop (Δimp, Ω, initial impedance‐ minimal impedance), mean temperature (°C), temperature rise (Δtemp, °C; maximum‐ minimum temperature), force time integral (FTI, g・s), and the AI (a.u.). Percentage impedance drop (%Δimp) was defined as 100 × (initial impedance – minimal impedance)/initial impedance.

VISITAG settings were as follows: (1) respiratory sync: enable, (2) catheter stability range of motion <= 3 mm, (3) catheter stability duration >3 s, and (4) FOT >3 g for 25% of the time.

The CF data were recorded every 0.05 s during the RF application and stored in the CARTO system separately from AI data. The standard deviation of the CF (CF‐SD[g]) was calculated from this data.^7^

We analyzed the ablation points which met the target CF 5‐15 g excluding the esophagus lesions in the SR group. In the AF group, in addition to the above criteria, we excluded ablation points made during sinus rhythm (i.e., excluded ablation points made after sinus recovery for any reason).

### 2.4 Statistical analysis

All continuous variables are summarized by mean ± SD and were compared using unpaired *t‐tests* or median (interquartile range [IQR]) and compared using Mann‐Whitney U tests. Categorical variables were compared using chi‐square or Fisher exact tests. Pearson's correlation coefficient was used to analyze correlations between two continuous variables. For all analyses, a two‐sided *p*‐value of <.05 was considered to indicate statistical significance (R, version 4.1.2).

## 3 RESULTS

### 3.1 Baseline characteristics

Table 1 shows the baseline characteristics of patients in the current study. Four patients were non‐PAF patients who converted to SR (anti‐arrhythmic drugs or defibrillation) before ablation and included in the SR group. Conversely, there were 2 patients who were PAF but in AF during the procedure and were included in the AF group. An enlarged left atrium, decreased ejection fraction, and LA strain were observed in the AF group. There was no difference in extracardiac parameters between the two groups.

**TABLE 1:** Baseline characteristics.

	SR group	AF group	*p* value
	*n* = 30	*n* = 30	
Age	70.0 ± 8.3	66.0 ± 9.2	.82
Gender, male	21 (70%)	25 (83%)	.35
BMI	24.2 ± 3.9	25.2 ± 3.3	.3
Type of AF			
PAF, *n* (%)	26 (86.7)	2 (6.7)	
Non‐PAF, *n* (%)	4 (13.3)	28 (93.3)	<.001
LAD, mm	38.6 ± 6.5	44.5 ± 4.1	<.001
LA‐Ss	14.3 ± 7.5	9.0 ± 5.6	.009
LA‐Sa	8.2 ± 5.1	NA	
LVEF, %	66.7 ± 9.3	56.3 ± 14.7	.002
NT‐proBNP, ng/mL	430 ± 643.2	1043 ± 1072	.009
CHADS2 score	1[0,2]	1[0,3]	.36

*Note*: Continuous variables are expressed as mean ± standard deviation, and scores are expressed as median and [25, 75%]‐ile range.

Abbreviations: AF, atrial fibrillation; LAD, left atrial diameter; LAVI, left atrial volume index; LVEF, left ventricular ejection fraction; PAF, paroxysmal atrial fibrillation; SR, sinus rhythm.

### 3.2 Procedure results and acquired points

Successful EEPVI was achieved in all patients. Concomitant CTI ablation and SVC isolation were performed in 42 and 45 patients, respectively. Total left atrial dwelling time was shorter (92.1 min) in the SR group than in the AF group (111.1 min, *p* = .012). More than 70% of ipsilateral PVI was achieved with 1st pass isolation (Table 2). Acute PV reconnections were observed in 6 (5%) PVs in the AF group and in 5 (4.2%) PVs in the SR group. There was no statistical difference between the two groups (*p* = 1.0).

**TABLE 2:** Procedure results.

	SR group	AF group	*p* value
	*n* = 30	*n* = 30	
1st pass			
RPV	21 (70%)	25 (83.3%)	.36
LPV	24 (80%)	22 (77.3%)	.76
LA procedure time, min	92.1	111.1	.012
CTI ablation	16 (55.2%)	26 (86.7%)	.01
SVC isolation	22 (75.9%)	19 (63.3%)	.399

Abbreviations: AF, atrial fibrillation; CTI, cavotricuspid isthmus; LA, left atrium; LPV, left pulmonary vein; RPV, right pulmonary vein; SR, sinus rhythm; SVC, superior vena cava.

### 3.3 Ablation point parameters

We obtained 1654 ablation points in the SR group and 2120 ablation points in the AF group. In the AF group, 88 points were obtained during sinus rhythm and excluded. Of these points, 1618 points (97.8%) in the SR group and 1961 points (96.5%) in the AF group had mean CF within the target range and were included for further analysis. Percentage of target CF achievement did not differ between the two groups (*p* = .18).

**FIGURE 2:**
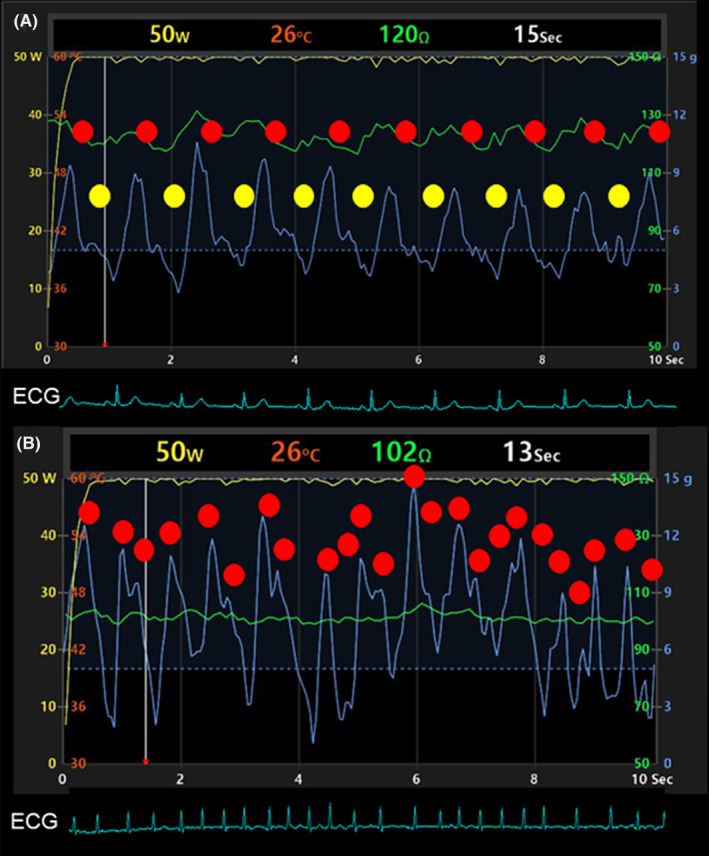
Example of contact force trace during radiofrequency application (RFA) for a patient in sinus rhythm (A) and a patient in atrial fibrillation (B). The blue line shows the contact force. The ECG shows lead 2. RF application was applied to the anterior segment of the right pulmonary vein in both rhythms. (A) RFA during sinus rhythm. Contact force shows a regular cyclic change. This change was created by the pump function (yellow circle) and the reservoir function (red circle). (B) RFA during AF rhythm. Contact force changed with beat‐by‐beat change of left atrial reservoir function (red circle) without pump function.

Table 3 shows ablation parameters. The mean CF (9.0 ± 2.4 vs. 9.1 ± 2.5 g), duration of RF application per point (11.6 ± 5.2 vs. 11.4 ± 5.1 s), mean power (48.3 ± 4.1 vs. 48.0 ± 4.3 W), FTI (101.6 ± 46.6 vs. 101.3 ± 45.6), and the AI (389.2 ± 58.6 vs. 387.5 ± 57.4) were not different between the SR group and AF group. The CF‐SD was 0.3 g (3.5 ± 2.2 vs. 3.8 ± 2.1 g, *p* < .001) higher and Δtemperature was 0.2°C (3.8 ± 1.3 vs. 4.0 ± 1.3°C, *p* < .001) higher in the AF group. On the other hand, Δimp (10.3 ± 5.8 vs. 9.4 ± 5.3 Ω, *p* < .05) and %Δimp (8.0 ± 3.9 vs. 7.3 ± 3.6%, *p* < .05) were higher in the SR group (Figure 2A,B).

**TABLE 3:** Ablation point parameters.

CF‐SD, g	SR group	AF group	*p* value
*n*	1618	1961	
Mean CF, g	9.0 ± 2.4	9.1 ± 2.5	.08
CF‐SD, g	3.5 ± 2.2	3.8 ± 2.1	.009
RF application time per point, s	11.6 ± 5.2	11.4 ± 5.1	.27
Mean power, W	48.3 ± 4.1	48.0 ± 4.3	.12
Δtemp, °C	3.8 ± 1.3	4.0 ± 1.3	<.001
FTI	101.6 ± 46.6	101.3 ± 45.6	.27
AI	389.2 ± 58.6	387.5 ± 57.4	.36
Δimp, Ω	10.3 ± 5.8	9.4 ± 5.3	<.001
%Δimp, %	8.0 ± 3.9	7.3 ± 3.6	<.001

Abbreviations: %Δimp, percentage delta impedance (100 × Δimpedance/initial impedance); AI, ablation index; CF, contact force; CF‐SD, standard deviation of contact force; FTI, force time integral; RF, radiofrequency; Δimp, delta impedance; Δtemp, delta temperature.

We did find no or weak correlation between CF stability and LAD (*r* = −.63, 95% CI −0.78 ~ −0.42, *p* < .05), LAVI (*r* = −.24, 95% CI −0.47 ~ −0.020, *p* = .07), and LA‐Ss (*r* = .05, 95% CI −0.42 ~ 0.33, *p* = .74).

### 3.4 Complications and follow‐up

No major complication associated with the ablation procedure was documented.

Five patients (16.7%) in the SR group and 2 patients (6.7%) in the AF group displayed documented AF (*n* = 5) or atrial tachycardia (*n* = 2) in the 8.3 ± 4 months of follow‐up (*p* = .24).

## 4 DISCUSSION

In this study, we performed PVI using a VIZIGO sheath. Using this visualized steerable sheath, we were better able to control the CF during RF application than for a conventional steerable sheath.^7^ Indeed, we selected patients for this study in whom this sheath and system had been used to minimize operator‐related contact force variability. Using the visualized steerable sheath, good catheter stability was obtained, and the size of the LAD, which seemed to affect the stability, did not correlate with the CF‐SD in this study.

The mean contact force was in the targeted range for more than 95% of ablation points in both groups, and there was no difference in mean CF between the two groups. RF application was uniformly performed at 50 W. RF application time per point did not differ between the two groups either. Under these circumstances, we achieved the same AI value in both groups. In theory, similar AI should indicate a similar lesion profile.^12^

However, there were three parameters related to lesion profile that differed between the two groups, and they were (1) CF‐SD, (2) impedance (Δimp and %Δimp), and (3) Δtemp.

### 4.1 Contact force stability during RF application

We used the standard deviation of CF to quantify CF stability. SD‐CF was greater for AF (*p* < .01). Respiration and LA contraction affect CF during RF application as intrinsic patient factors. In this study, all patients breathed spontaneously under conscious sedation with opioids, so we consider respiration to have been similar for the two groups. Left atrial movement can be measured by transthoracic echocardiogram as LA‐Ss representing LA reservoir function and LA‐Sa representing pump function^13^ during SR. Although LA reservoir function remains^14^ during AF rhythm, it does vary according to the previous RR interval.^15^ Also during AF, pump function is lost. CF is affected by LA movement because the catheter is in contact with the LA wall. We believe the irregular wall motion of the LA during AF produces the greater CF‐SD. The absolute value of the difference between the two groups in CF‐SD was 0.3 g. This may seem trivial, but in the low CF‐based HPSD situation, the relative difference in CF‐SD corresponded to a more than 3% difference.

### 4.2 Greater impedance drop in SR

Previous reports showed that AI was associated with impedance drop and %imp.^1^, ^16^ The Δimp and %Δimp were higher in the SR group than in the AF group, despite having the same AI value. CF‐SD in this study was smaller in the SR group. We therefore hypothesize that higher Δimp and %Δimp in the SR group were due to better CF stability. To create an optimal lesion, a larger Δimp would be preferable given the same AI setting, which is one of the reasons we believe RF application should be performed during SR.

Although there is one study finding that the generator impedance was associated with local myocardial tissue damage when compared to local impedance,^16^ other studies have described an association between generator impedance and ablation lesion.^17^ Our current results suggest that generator impedance does affect the ablation lesion.

### 4.3 Ablation catheter tip temperature rise

The method of radiofrequency ablation widely performed these days is power‐controlled ablation with open‐irrigated ablation catheters. Tip temperature during RFA alone is not the most important factor we attend to because it does not predict lesion size and steam pop.^4^

Considering that the Δimp of the SR group was greater than that of the AF group, it is expected that the RF energy given to the myocardium was higher in SR. The atrial function was reduced in this study during AF^18^, ^19^, ^20^ as previously reported, and blood flow was more likely to be slow in the atria than in SR, thus preventing the dissipation of a given thermal energy from the ablation site.

### 4.4 Optimal rhythm for RF application

In this study, we evaluated the ablation point profile in AF and SR conditions. Under the same AI settings, more stable CF and higher impedance drop were obtained in the SR group compared to the AF group. The AI predicts the RF effect based on only 3 parameters, time, CF, and power, and does not reflect any other factors, in particular, atrial rhythm status. AI was calculated with a CF sampling frequency of 100 ms. Therefore, it did not reflect the variability of the CF caused by the atrial contraction which oscillates in a cycle shorter than 100 ms. Although it has been reported that AI correlates with lesion size in in vitro models,^12^ the cardiac rhythm during RF application can be considered as one of the limitations of AI‐guided ablation in clinical use. In general, the interpretation of the local electrogram acquired by ablation catheter is superior during SR. Ideally, RF application during SR is desirable. However, the benefit has to be weighed against the burden of repeated defibrillation to achieve SR, especially in cases of longstanding persistent AF. In such cases, RF application should be performed while keeping in mind that the RF effect is inferior during AF compared to SR.

A previous study demonstrated how ablation parameters varied due to endogenous factors in the same way as this study.^21^ Neither study used deep sedation or general anesthesia. However, our study differed from the previous one in the following aspects: (1) CF stability was defined as the average of the standard deviation of the CF, while the previous study defined it as the difference between the average peak value and the average trough value, (2) the previous study was from the low‐output era, so the ablation time was longer with 30 W output, and (3) the previous study investigated the impedance changes for each FTI, while this study used a fixed AI as the RF target. As a result, the previous study reported less variation in CF during AF. By using a visible sheath and the HPSD strategy, this study expected the peak difference of CF to be smaller due to the lower CF and shorter ablation time, so it used the average and the error from it as the indicator of CF stability. In our study, AI around 390 corresponded to FTI around 100, and the previous study's graph showed no difference in impedance change between SR and AF at the point of FTI100. This study demonstrated the differences in energy efficiency in the HSPD era.

### 4.5 Limitation

We were able to find differences between SR and AF in items that are surrogate markers of the RF application effect, but not in hard endpoints such as 1st pass success rates and durability of PVI. This might be subjected to the skill of the surgeon and the fact that a high 1st pass rate and low acute PV reconnection rate were already achieved in both groups.

Another limitation of this study was its retrospective design. However, even with a prospective study, it was not possible to blind the rhythm during radiofrequency application. Therefore, in this study, we believe that we were able to analyze the real‐world radiofrequency effect by examining consecutive cases using a visualized steerable sheath.

## 5 CONCLUSION

Despite similar AI, various measures of RF application differed between that delivered during SR and AF. CF was more variable during AF and impedance drop was smaller, suggesting less effective lesion formation in AF. Such differences may be useful to keep in mind during PVI.

## FUNDING INFORMATION

Not applicable.

## CONFLICT OF INTEREST STATEMENT

The authors declare no conflicts of interest.

## ETHICS STATEMENT

Not applicable.

## PATIENT CONSENT STATEMENT

Not applicable.

## CLINICAL TRIAL REGISTRATION

Not applicable.

## DATE AVAILABILITY STATEMENT

The data that support the findings of this study are available from the corresponding author upon reasonable request.
